# Supplementary material for: Structural basis of transcriptional regulation by a nascent RNA element, HK022 putRNA
Source: Nat Commun. 2022 Aug 15;13:4668. doi: 10.1038/s41467-022-32315-y (PMC9378689; doi:10.1038/s41467-022-32315-y)
Supplement: Supplementary file 1 — Supplementary Information [file 41467_2022_32315_MOESM1_ESM.pdf]

Supplementary Note 1 Optimization of DNA scaffold regarding the distance between the pausing site and the *lacO* sequence for *put*EC preparation

To construct a DNA scaffold that stalls *put*EC at the native pausing site where the EC pauses in the absence of an intact *put*RNA, we modeled an EC and a LacI tetramer bound on a *lacO* sequence by using deposited PDB files (PDB 1EFA (LacI), 6ALF (EC)) and found 10-nt spacer is the shortest one that brings *lacO*-bound LacI tetramer and an EC close without making clashes between the two proteins (Supplementary Fig. 1a). However, the 10-nt spacer-containing DNA scaffold produced a longer RNA than the originally paused RNA (Supplementary Fig. 1b). Therefore, we designed DNA scaffolds having 6 to 9 spacers, performed transcription assays with the scaffolds, and chose to use a 7-nt spacer-containing scaffold (named 7-nt) that produces stalled RNA that is most similar to the paused EC with *put⁻* sequence (Supplementary Table 1).

Supplementary Table 1 The DNA sequences of the scaffolds used for screening

Name	DNA Sequence
6-nt	GCATGAATTCCTATTGGTAC<u>TTTACA</u>TTAATGAACTTTAAGTA<u>CATCAT</u>AAGCCC**A**TAGACGAACGGCGCGTCTTTAAACCATGCGTCGGGAGCGCGGCGGGTTCAGGATGAACGGCAATGCTGCTCATTAGCGAGAAGGCTTTTTT**G**CTTTTAAATTGTGAGCGCTCACAATTCG
7-nt	GCATGAATTCCTATTGGTAC<u>TTTACA</u>TTAATGAACTTTAAGTA<u>CATCAT</u>AAGCCC**A**TAGACGAACGGCGCGTCTTTAAACCATGCGTCGGGAGCGCGGCGGGTTCAGGATGAACGGCAATGCTGCTCATTAGCGAGAAGGCTTTTTT**G**CTTTTAGAATTGTGAGCGCTCACAATTCG
8-nt	GCATGAATTCCTATTGGTAC<u>TTTACA</u>TTAATGAACTTTAAGTA<u>CATCAT</u>AAGCCC**A**TAGACGAACGGCGCGTCTTTAAACCATGCGTCGGGAGCGCGGCGGGTTCAGGATGAACGGCAATGCTGCTCATTAGCGAGAAGGCTTTTTT**G**CTTTTAGTAATTGTGAGCGCTCACAATTCG
9-nt	GCATGAATTCCTATTGGTAC<u>TTTACA</u>TTAATGAACTTTAAGTA<u>CATCAT</u>AAGCCC**A**TAGACGAACGGCGCGTCTTTAAACCATGCGTCGGGAGCGCGGCGGGTTCAGGATGAACGGCAATGCTGCTCATTAGCGAGAAGGCTTTTTT**G**CTTTTAGTCAATTGTGAGCGCTCACAATTCG
10-nt	GCATGAATTCCTATTGGTAC<u>TTTACA</u>TTAATGAACTTTAAGTA<u>CATCAT</u>AAGCCC**A**TAGACGAACGGCGCGTCTTTAAACCATGCGTCGGGAGCGCGGCGGGTTCAGGATGAACGGCAATGCTGCTCATTAGCGAGAAGGCTTTTTT**G**CTTTTAGTCAAATTGTGAGCGCTCACAATTCG

-35 element and -10 element are underlined. The transcription start site is colored in blue, and the native pausing site is in red. The *lacO* sequence is colored in green

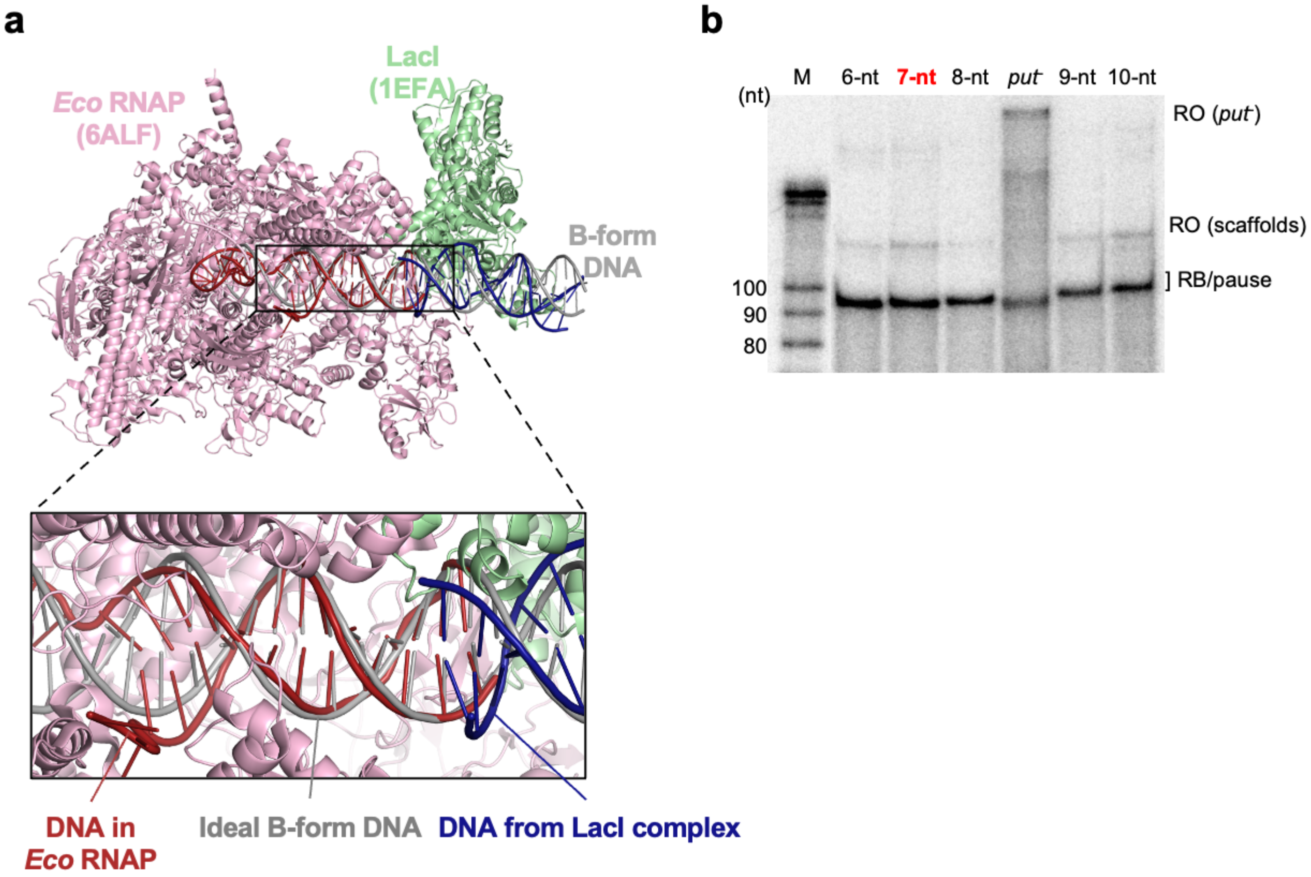

Supplementary Fig. 1 Design of *put*EC DNA scaffolds. a For the DNA scaffold design, an EC structure (PDB 6ALF) was first placed on an ideal B-form DNA by overlapping the downstream DNA of the EC with the ideal B-form DNA. Then, the LacI tetramer (PDB 1EFA) was placed on the B-form DNA downstream to the EC by aligning the *lacO* DNA of the LacI complex to the B-form DNA. Then, the LacI model was shifted along the B-form DNA toward EC until the *Eco* RNAP and the LacI touch each other. This modeling revealed that the shortest spacer that does not make clashes between the two proteins was 10-nt. However, the *lacO* sequence, as well as downstream DNA in an EC, are not straight like an ideal B-form DNA, therefore, the minimal spacer length can be different from the model. In addition, RNAP might tolerate some clashes because the RNAP domains touching the LacI are flexible. **b** Radiolabeled transcription assay was performed with designed scaffolds (Supplementary Table 1) in the same method as Fig. 1c. Details are written in Methods.

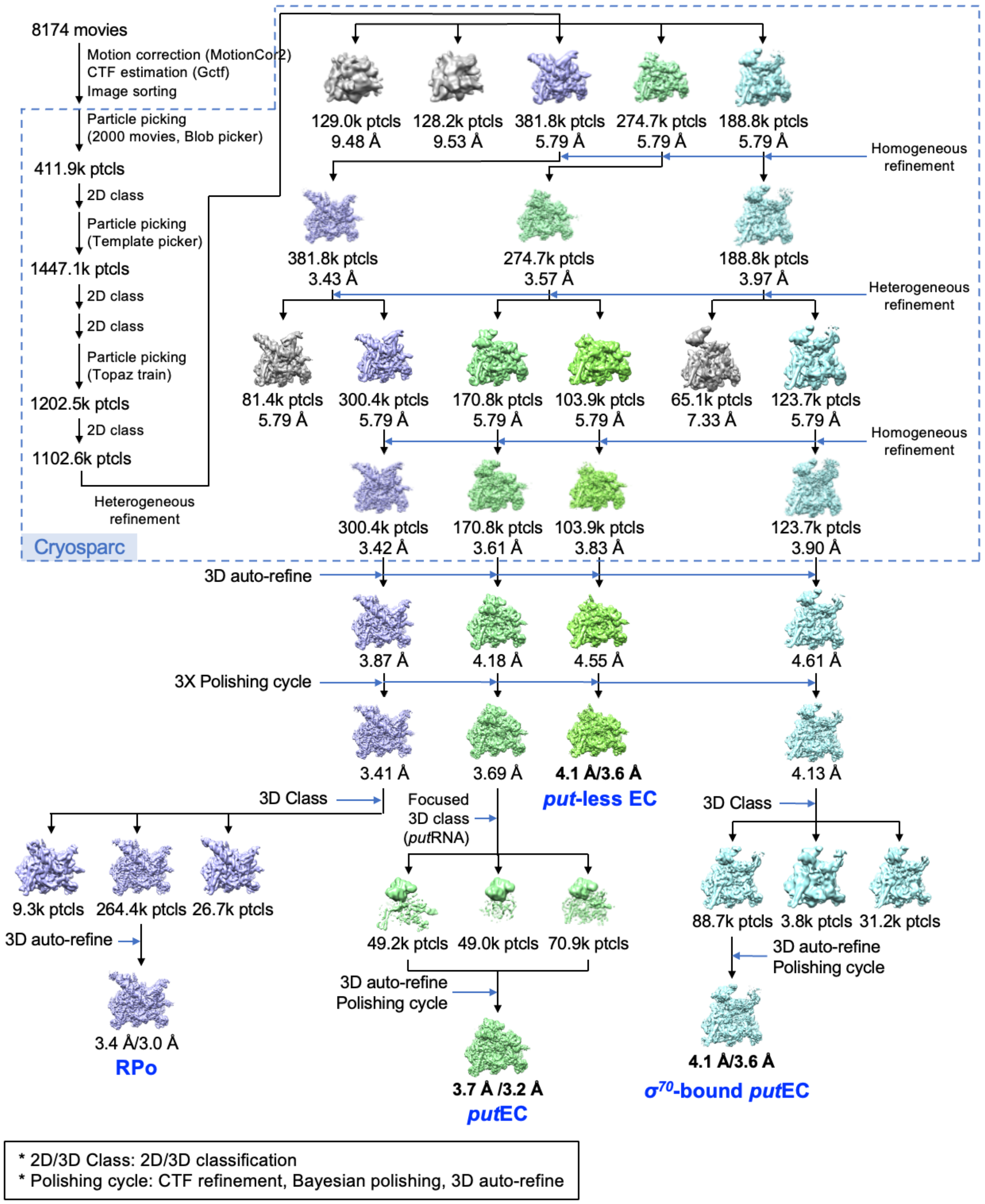

Supplementary Fig. 2 Flowchart for cryo-EM data analysis of *put*EC. Total 8174 movies of *put*EC were motion-corrected and summed by MotionCor2[1], CTF-estimated by Gctf [2] and sorted by the CTF parameters in RELION3.1[3]. The sorted images were transferred to cryoSPARC v3.2.0[4] for further process as follows: First, 411.9k particles were picked using blob picker from 2000 movies, extracted with 320 pixels box size, and 2D classified to make picking templates. Then, 1447.1k particles were picked using template picker from 8174

images. The particles were 2D classified twice, and the selected 863.1 k particles from 43 classes were used as templates for Topaz picker [5]. From Topaz train, 1202.5k particles were picked and extracted from 8162 images. The particles were 2D classified into 100 classes and 90 classes were selected. The selected particles were divided into five classes in heterogeneous refinement. Among the five templates, three are from the previous dataset collected from Glacios, two are from EMDB EMD-8585, an electron density map of non-paused EC. Among five classes, three classes were subjected to homogeneous refinement. Each homogeneous-refined class was further heterogeneous-refined into two classes, resulting in total four significant classes – RPo, *put*EC, *put*-less EC, and σ^{70}-bound *put*EC.

All particles of the four classes were imported to RELION3.1 for further refinements. The particles in RPo class were 3D auto-refined, particle-polished three times, and 3D-classified into three classes. Among the three classes, the major class was 3D auto-refined and postprocessed yielding 3.0-Å resolution map. The *put*EC particles were 3D auto-refined, particle-polished three times, and subjected to focused classification onto *put*RNA region into three classes. Among the three classes, two classes are combined, 3D auto-refined and post-processed yielding 3.2 Å-resolution map. The *put*-less particles were 3D auto-refined, particle-polished three times, and post-processed yielding 3.6 Å-resolution map. The σ^{70}-bound *put*EC particles were 3D auto-refined, particle-polished three times, and 3D classified into three classes. Among the three classes, one best class was further refined and post-processed yielding 3.6 Å-resolution map.

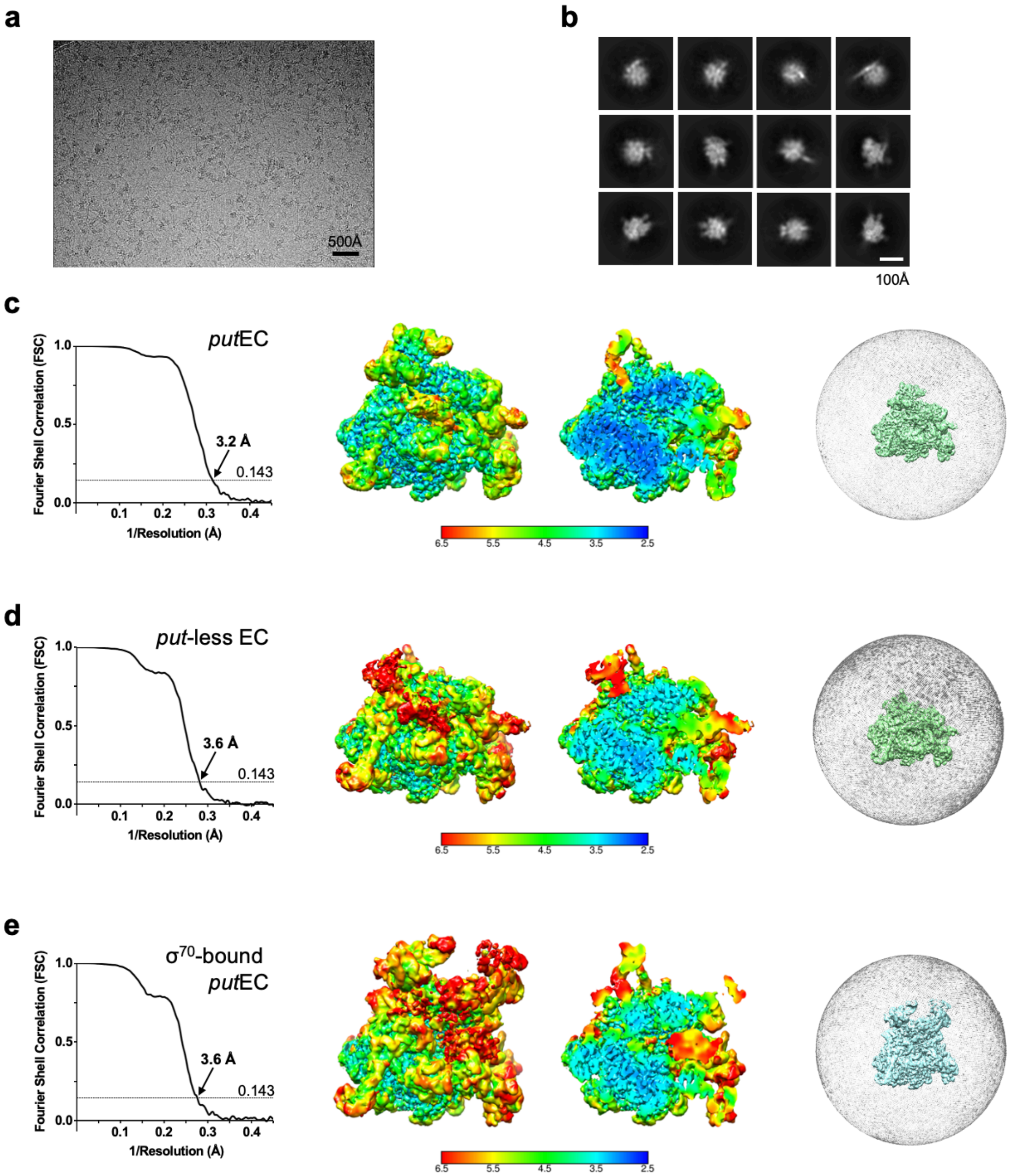

Supplementary Fig. 3 Cryo-EM map analysis of the *put*EC. a A representative cryo-EM image. **b** Representative 2D classes. **c** FSC curve (left), a whole and a half-cut local resolution map (middle), and angular distribution of the particle projections of the *put*EC. **d** FSC curve (left), a whole and a half-cut local resolution map (middle), and angular distribution of the particle projections of the *put*-less EC. **e** FSC curve (left), a whole and a half-cut local resolution map (middle), and angular distribution of the particle projections of the σ^{70}-bound *put*EC.

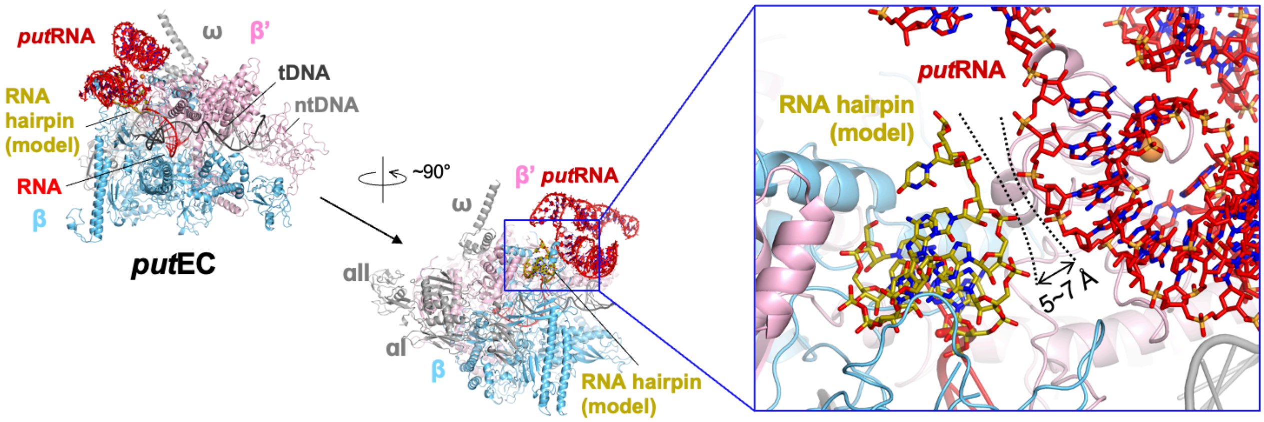

Supplementary Fig. 4 Modeling of an RNA hairpin in the RNA exit channel in the *put*EC. An RNA hairpin is modeled based on the *his*PEC structure (PDB 6ASX). The phosphate backbone of the modeled RNA hairpin is only 5-7 Å away from the phosphate backbone of the *put*RNA, implying that electrostatic repulsion would occur between the RNA hairpin and the *put*RNA.

Supplementary Note 2 Rationale of de novo building of *put*RNA in the cryo-EM density

The local resolution of *put*RNA region is ~3.5 Å, which is not sufficient to differentiate RNA bases (Adenine, Cytosine, Uracil, and Guanine). However, the predicted two stem-loop structures and the potential base-pairs from the sequence specify the 3-dimensional structure of HK022 *put*L RNA. Here is how we build the RNA in order:

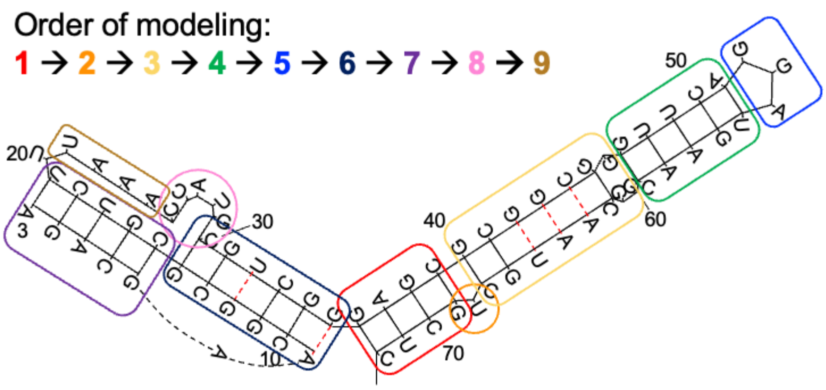

1. Building stem II region in HK022 *put*

1. The 3'-end of the RNA was identified by the density connecting the *put*RNA and the RNA-DNA hybrid in the RNAP.
2. The predicted 14-bp A-form RNA duplex structure was roughly placed in the stem II region (Supplementary Fig. 6b). The model was prepared by using 'Other Modeling Tools' in Coot.
3. Then, the 3'-end part of the stem II model (C72*-G69*:G36*-C39*) was finely fitted to the map. This revealed the location of the unpaired base, U68*, because it is wedged in the middle of RNA duplex, bending the stem II.
4. After placing U68*, the middle part of the stem II was modeled. Here, the unexpected, three continuous non-canonical base pairs (G42*-C44*:U65*-A63*) were revealed. During modeling, we were assured of the location of these bases due to the clear density of the phosphate backbone. The following unpaired bases, G46*, G60*, and G61*, could be located according to the continuous phosphate backbone density as well.
5. The rest region of the stem II (G47*-C59*), which contains a 5-bp stem with a 3-nt loop, was built in the rest part of the map. This region has a relatively low local resolution. However, this region is a canonical A-form RNA duplex and the map is good enough for placing an RNA duplex.

2. Building stem I region of HK022 *put*

1. Although G35* was predicted to be unpaired, there was no unpaired nucleotide between stem I and stem II. Thus, an RNA duplex that contains the predicted 8-bp and one additional base pair (G35*-A9*) was placed in the stem I region. Interestingly, there was an additional density around the stem I structure.
2. The base pairs were manually examined from the bifurcation point G35*-A9*. Interestingly, although RNA duplex structure was maintained, the phosphate backbone was broken between the C30* and the next nucleotide in the stem I. In addition, A9* nucleotide was

connected to the density from prior nucleotides. We built the bases A8*, G7*, C6*, A5*, G4*, and A3* and found that these nucleotides participate in the stem I duplex, particularly, forming duplex following C15*-U19*.

3. We modeled nucleotides along the phosphate backbone upstream of C30*. G29*, U28*, A27*, and C26* formed a bulge, and the U28* base was binding to a pocket formed with RNAP β' residues, L71, P51, R53, and R60. This pocket is adjacent to the β'ZBD.

4. Then the upstream part of the bulge (C26*, C25*, A24*, A23*, A22*, U21*, and U20*) was placed in the minor groove of the stem I duplex. Now all the density for HK022 *put* was filled and the base pairs in this unexpected structure made sense as shown in Fig. 2c.

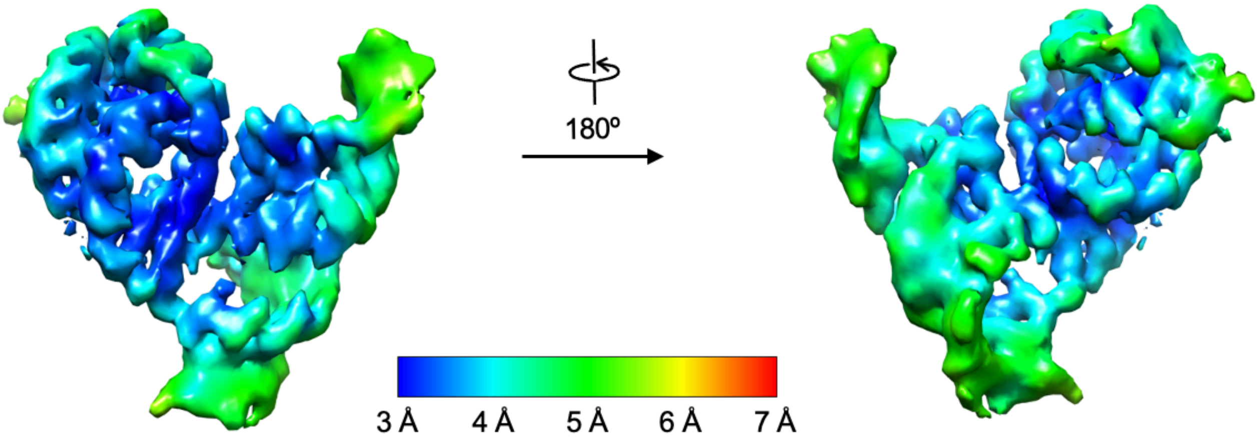

Supplementary Fig. 5 Local resolution map of the *put*RNA. Local resolution of the *put*RNA region is displayed on the cryo-EM map of *put*RNA. For clarity, the remaining part of the map is omitted.

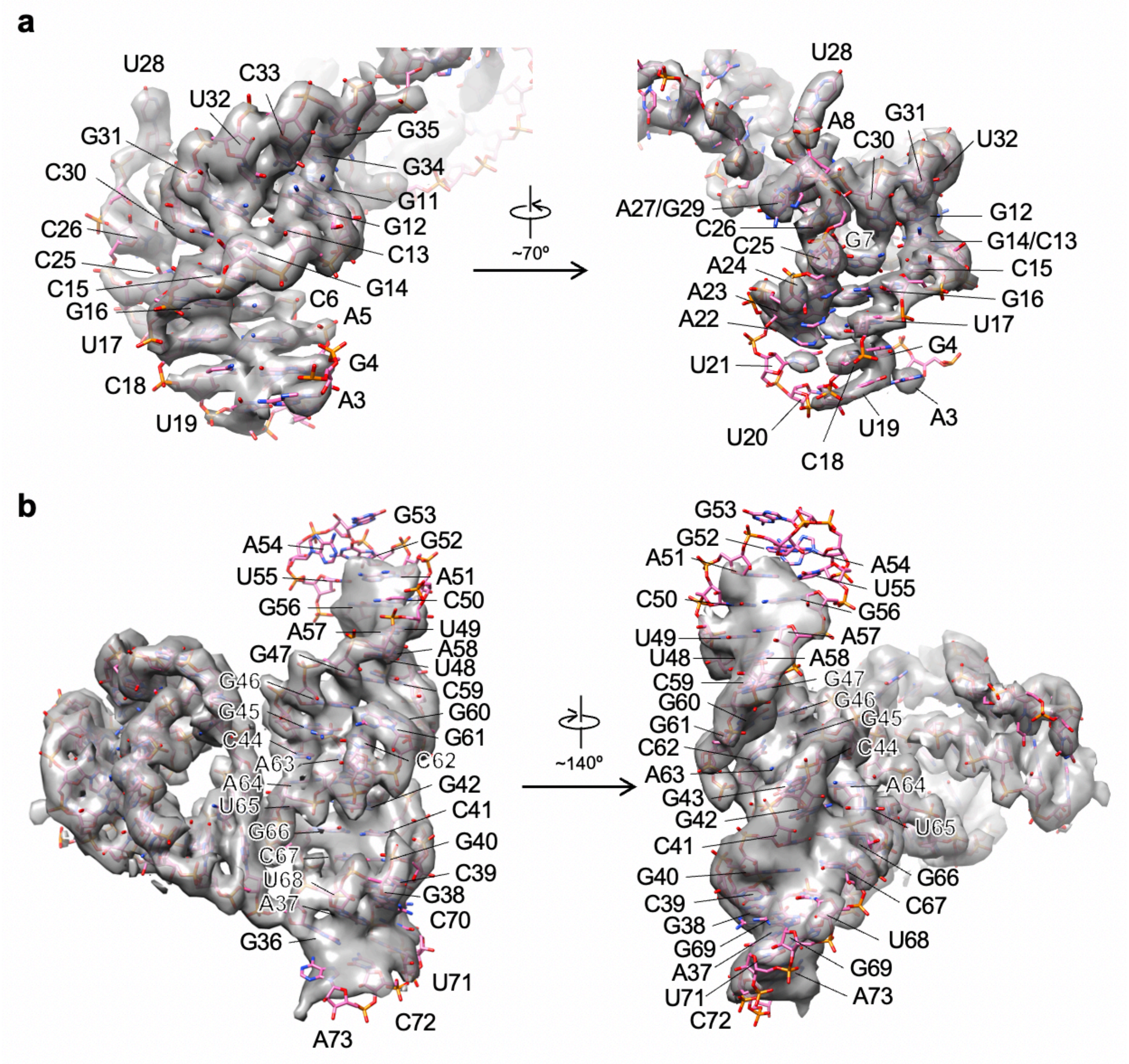

Supplementary Fig. 6 Close-up views of the *put*RNA map and model. Cryo-EM density of *put*RNA is drawn by a semi-transparent surface format and the modeled *put*RNA structure is superimposed. Most residues are labeled. **a** A view for stem I region. **b** A view for stem II region.

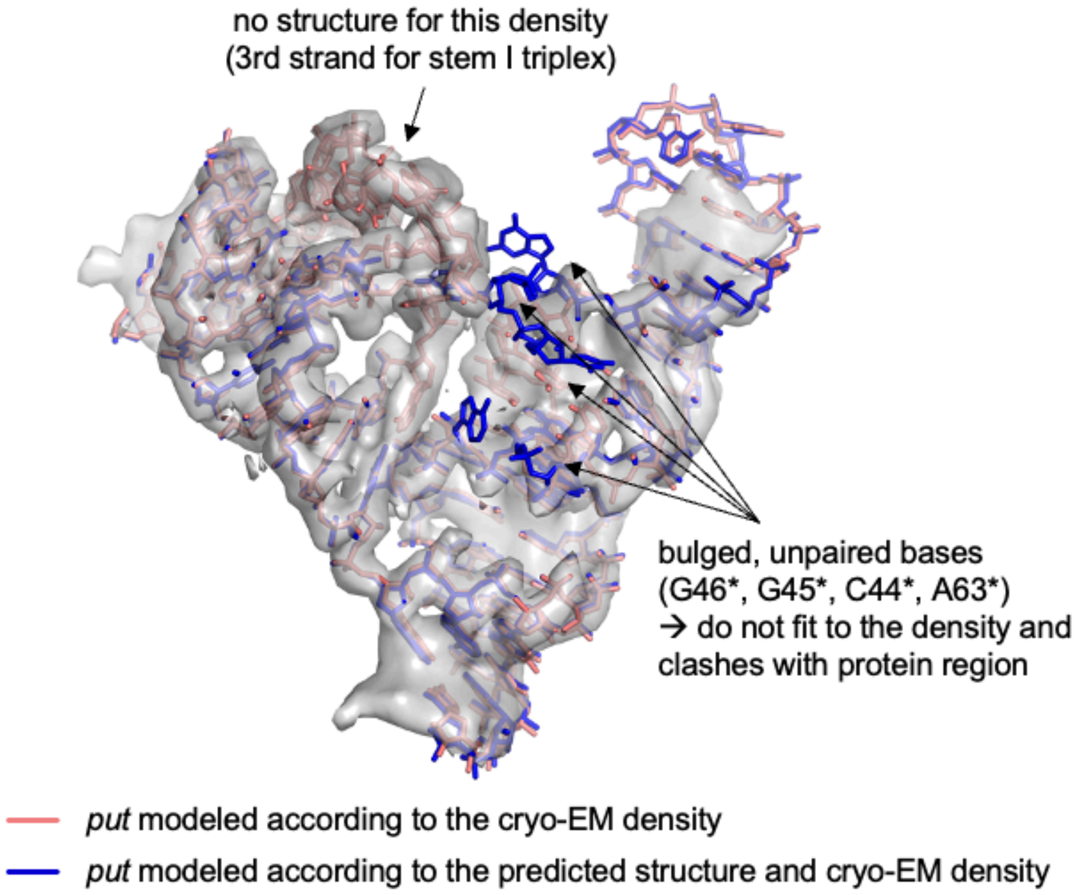

Supplementary Fig. 7 Comparison of the *put*RNA model built on the cryo-EM map and the *put*RNA model built by the predicted structure. After we modeled the *put*RNA structure according to the cryo-EM map, we modified the *put*RNA structure according to the predicted structure trying to fit it into the cryo-EM map. Then, the two structures were superimposed onto the cryo-EM map. Cryo-EM density of *put*RNA is drawn by a semi-transparent gray surface format, and the modeled *put*RNA structures were drawn in stick format in red and blue, as the labels in the figure indicate. The predicted structure does not fill the third strand density in the stem I region and exhibits bulged and unpaired bases on the interface between the *put*RNA and the RNAP, implying that the predicted model is not correct.

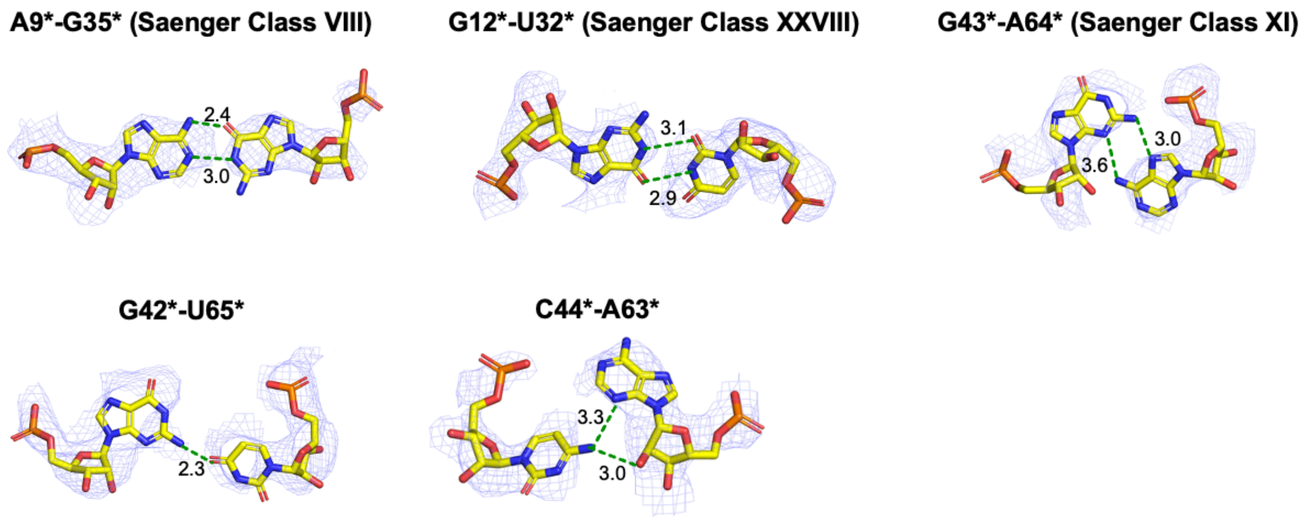

Supplementary Fig. 8 Non-canonical base pairs in *put*RNA. The non-canonical base pairs of the *put*RNA are drawn in stick format and the map densities for the base pairs are superimposed in mesh format. Green dotted lines represent hydrogen bonds, and the length of each bond is written nearby the line in Å unit.

Supplementary Table 2 The potential salt bridges and hydrogen bonds between the *put*RNA and the RNAP. The local resolution of the *put*RNA region is ~3.5 Å, which is relatively low to define short-distance range interactions such as salt bridges and hydrogen bonds. However, to describe the potential interactions on the interface between the *put*RNA and the RNAP, potential salt bridges and hydrogen bonds are calculated by PDBePISA[6] and listed below after the map inspection. The list is divided by the interfaces with β'ZBD (#1-#13) and βFTH (#14-#16).

Number	*put*RNA residue	*put*RNA atom	RNAP residue	RNAP atom	Distance (Å)	Kind	Info
1	A8	O2'	β'K87	NZ	2.9	H-bond	
2	A8	O3'	β'K87	NZ	3.0	H-bond	β'K87-NZ is shared
3	A9	OP1	β'K87	NZ	2.6	Salt bridge	
4	A9	N6	β'K74	O	2.7	H-bond	A9-N6 is shared
5	A9	N6	β'G73	O	3.2	H-bond	
6	U28	O4	β'A59	N	3.0	H-bond	
7	C33	OP1	β'K66	NZ	2.9	Salt bridge	
8	G34	O6	β'K76	NZ	3.1	H-bond	
9	G35	O2'	β'R77	NH1	2.5	H-bond	
10	A63	O2'	β'Y75	OH	3.0	H-bond	
11	A64	OP1	β'K79	NZ	2.4	Salt bridge	A64-OP1 is shared
12	A64	OP1	β'H80	NE2	2.9	H-bond	A64-OP1 is shared
13	U65	OP1	β'R77	NH2	2.2	Salt bridge	
14	G11	N2	βQ894	OE1	3.1	H-bond	The local resolution of this β loop is ~3.5 - 4.5 Å
15	U32	O2'	βE892	O	3.0	H-bond	
16	G34	O4'	βQ894	NE2	3.6	H-bond	

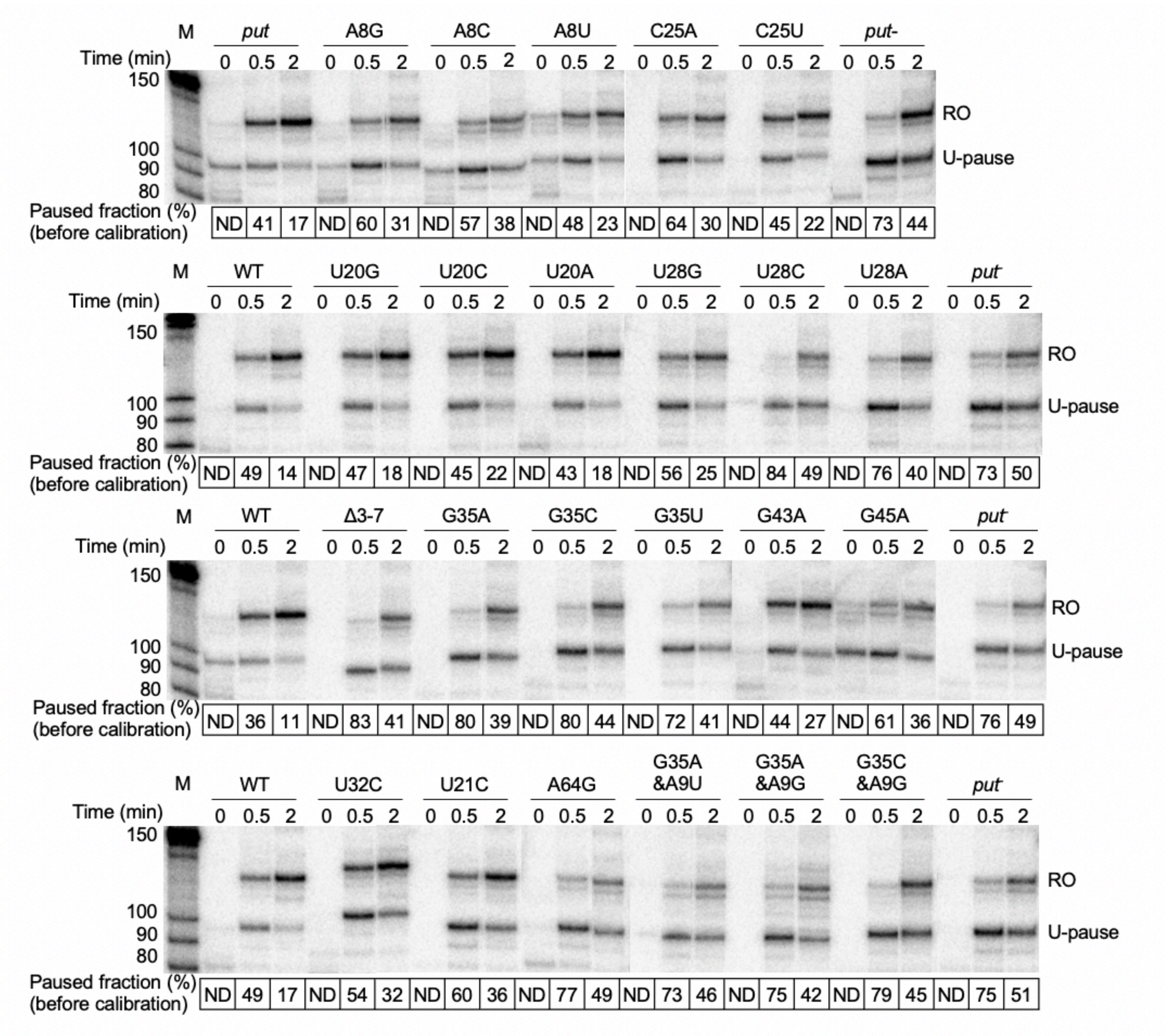

Supplementary Fig. 9 Anti-pausing activity measurement of *put*RNA mutants by radiolabeled transcription assay. Radiolabeled transcription assay was performed with the *put* mutants in the absence of roadblock protein LacI to measure the anti-pausing activity of the mutations in triplicate. In the assay, 50 nM holoenzyme and 12 nM template DNA were mixed to form an open complex in the reaction buffer (20 mM Tris-glutamate pH 8.0, 150 mM K-glutamate, 10 mM Mg-glutamate, 5 mM DTT). 200 μM ATP, 200 μM UTP, 200 μM GTP, 25 μM CTP and 0.05 μM α-^{32}P CTP were added to initiate transcription, and the reaction was quenched at 0-, 0.5-, and 2-min time points by adding 2X loading buffer containing 10 M urea. For the quantification of anti-pausing activity, the data at 0.5 min were used while 2-min data produced similar result. The anti-pausing activities were calculated by the equation shown in the Methods section, and plotted in Fig. 3c. The gel image above is from one of the triplicate experiments.

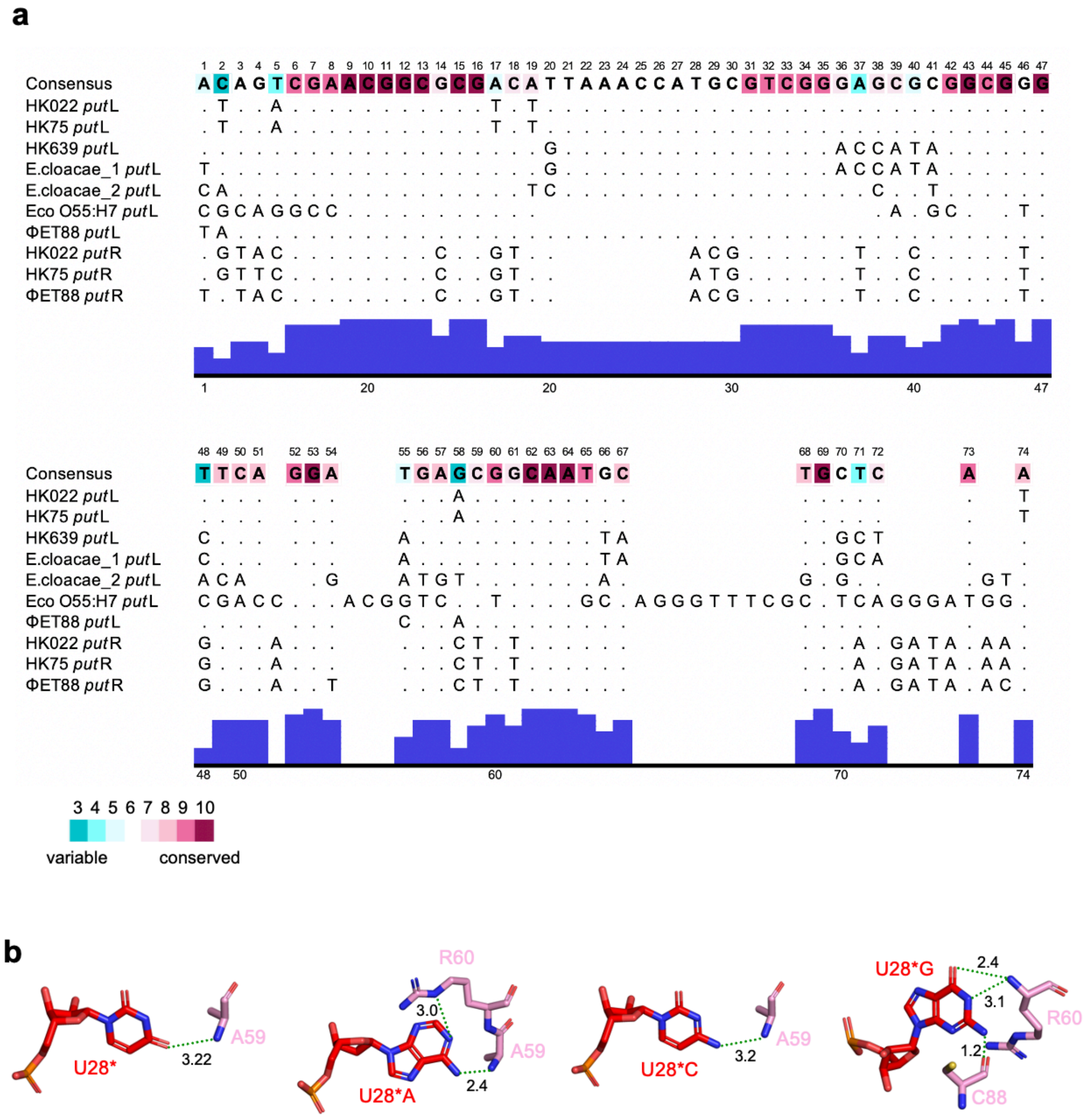

Supplementary Fig. 10 Multiple sequence alignment of *put* and modeling of U28* substitution. a Ten *put*RNA sequences from different species are aligned in LocARNA server[7,8], and the sequence conservations were scored. The conservation score is shown in color. **b** Structures of U28* and modeled structures of U28* mutations (U28*A, U28*C, U28*G) are drawn with their interactions with surrounding β’ residues. The potential hydrogen bonds are drawn as green dotted lines. The length of each bond is written nearby the line in Å unit.

Supplementary Table 3 Global conformational change comparing the *put*EC and other EC structures. The various EC structures including non-paused EC (PDB 6ALF, marked as EC), *his*PEC (PDB 6ASX), backtracked PEC (PDB 6RIP, marked as btPEC), *put*EC, *put*-less EC, σ^{70}-bound *put*EC (or σ-*put*EC), were aligned by core module and the RMSD values between the domains of the ECs and those of the *put*EC were calculated. The largest RMSD value in each domain is colored in light pink and the smallest RMSD value is colored in light green. The RMSD values between *put*EC and σ^{70}-bound *put*EC are calculated but excluded from the comparison because the σ^{70}-bound *put*EC is in an open clamp state, therefore, intrinsically different from all the other EC structures. The comparison indicates that the *put*EC structure is the most similar to the non-paused EC structure, and the most different from the *his*PEC. Standard error is calculated from the RMSD values of the four comparisons (*put*EC-EC, *put*EC-*his*PEC, *put*EC-*put*-less EC, and *put*EC-btPEC). (For the calculation, the rms_cur command was used in PyMOL to include the change in the domain location after all the EC structures were aligned by the core module)

Structural Module	Range	Total # of C_α	RMSD (Å)						Standard deviation
			*put*EC-EC	*put*EC-*his*PEC	*put*EC-btPEC	*put*EC-*put*-lessEC	*put*EC-σ-*put*EC	EC-*his*PEC	
Entire Structure	All	3153	1.004	2.424	1.850	1.874	5.293	2.482	0.586
Core	β: 3-30, 140-150, 445-455, 513-713, 786-832, 1056-1295<br>β': 343-786<br>ω: all	1054	0.610	0.655	0.588	0.486	0.821	0.776	0.071
β1-lobe (protrusion)	β: 31-139, 456-512	166	1.008	1.157	1.357	0.905	1.301	1.529	0.196
β2-lobe:SI1	β: 151-444	294	1.175	1.390	1.084	1.157	1.490	1.656	0.132
βflap:SI2	β: 833-1055	198	0.894	5.775	3.063	5.534	5.741	6.121	2.302
Clamp	β: 1319-1341<br>β': 16-342, 1318-1344	350	1.560	3.121	2.382	1.747	14.767	3.021	0.706
β'dock	β': 369-420	52	0.618	1.209	0.888	0.781	1.966	1.392	0.249
β'shelf/β'jaw	β': 787-931, 1135-1317	327	1.073	3.127	2.707	2.135	1.890	2.648	0.890
β'SI3	β': 948-1121	174	1.543	4.527	4.146	2.815	2.388	4.760	1.358
Clamp/ β'dock/shelf/ jaw/SI3/ β'C	β: 1241-1341<br>β': 16-342, 369-420, 787-931, 948-1121, 1135-1373	1037	1.294	3.178	2.702	1.978	8.791	3.071	0.826

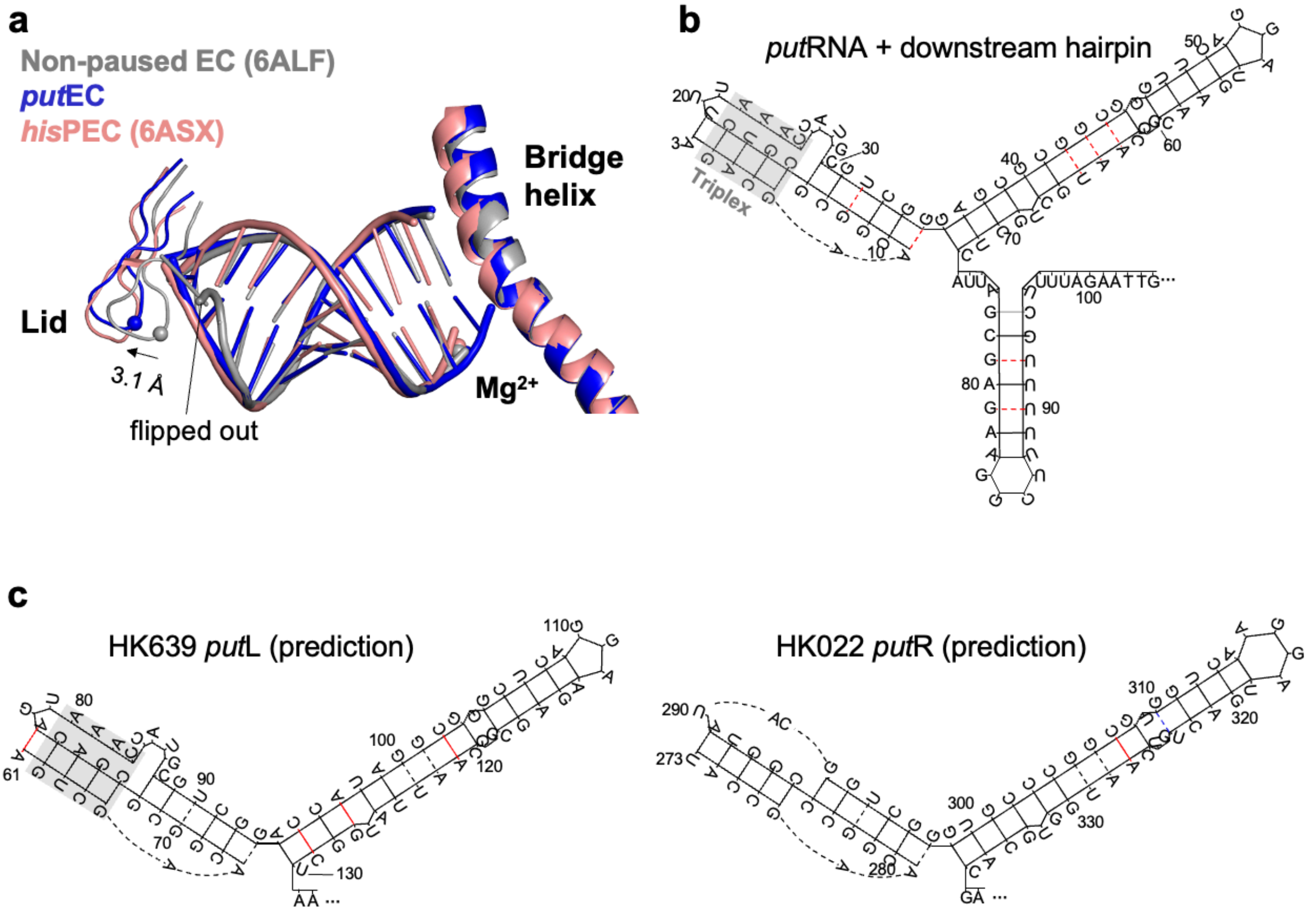

Supplementary Fig. 11 RNA-DNA hybrids in ECs and diverse *put*RNA models. a RNA-DNA hybrids and the structural elements next to the hybrid – lid and bridge helix – are drawn. In the previously determined EC structures, RNA-DNA hybrids are 10-nt long[9–12]. However, the lid, which helps to separate RNA-DNA hybrid and rewind the upstream DNA duplex, is pushed further in *his*PEC probably due to the half-translocated RNA-DNA hybrid in *his*PEC. Meanwhile, in the *put*EC which contain 11-nt RNA-DNA hybrid, the lid is located similar with that of *his*PEC, and its lid is displaced by 3.1 Å from that of the non-paused EC (measured between the C_α atoms of β'D256). The figure also shows that the RNA residue located at the *i-10* site in non-paused EC is flipped out. **b** The *put*RNA sequence containing potential downstream RNA hairpin structure was predicted from the RNAfold Server[13]. In the σ^{70}-bound *put*EC map, downstream duplex RNA is observed in the RNA exit channel. The RNA model was built according to the map and the first potential A-U base pair in the downstream RNA hairpin was not observed, therefore, the base pairing was not indicated in the figure. **c** Structures of HK639 *put*L and HK022 *put*R are modeled based on the sequence alignment and the cryo-EM structure of HK022 *put*L. In both structures, many base-pairs are conserved except the ones marked with red lines.

Supplementary Table 4 Global conformational change comparing the *put*-less EC and other EC structures. The various EC structures including non-paused EC (PDB 6ALF, marked as EC), *his*PEC (PDB 6ASX), backtracked PEC (PDB 6RIP, marked as btPEC), *put*EC, *put*-less EC, σ^{70}-bound *put*EC (or σ-*put*EC), were aligned by core module and the RMSD values between the domains of the ECs and those of the *put*-less EC were calculated. The largest RMSD value in each domain is colored in light pink and the smallest RMSD value is colored in light green. The RMSD values between *put*-less EC and σ^{70}-bound *put*EC are calculated but excluded from the comparison because the σ^{70}-bound *put*EC is in an open clamp state, therefore, intrinsically different from all the other EC structures. The comparison indicates that the *put*-less EC structure is the most similar to the *put*EC structure, and the most different from the non-paused EC. Standard deviation is calculated from the RMSD values of the four comparisons (*put*-less EC-EC, *put*-less EC-*his*PEC, *put*-less EC-btPEC, and *put*-less EC and *put*EC). (For the calculation, the rms_cur command was used in PyMOL to include the change in the domain location after all the EC structures were aligned by the core module)

Structural Module	Range	Total # of C_α	RMSD (Å)						Standard deviation
			put-less EC-EC	*put*-less EC-*his*PEC	*put*-less EC-btPEC	*put*-less EC-*put*EC	*put*-less EC-σ-*put*EC	EC-*his*PEC	
Entire Structure	All	3153	1.997	1.237	1.456	1.004	5.023	2.482	0.324
Core	β: 3-30, 140-150, 445-455, 513-713, 786-832, 1056-1295<br>β': 343-786<br>ω: all	1054	0.681	0.539	0.521	0.610	0.635	0.776	0.086
β1-lobe (protrusion)	β: 31-139, 456-512	166	1.396	0.946	1.252	1.008	1.079	1.529	0.238
β2-lobe:SI1	β: 151-444	294	1.516	1.203	1.230	1.175	1.094	1.656	0.162
βflap:SI2	β: 833-1055	198	5.845	2.469	4.209	0.894	1.898	6.121	1.537
Clamp	β: 1319-1341<br>β': 16-342, 1318-1344	350	1.929	1.647	1.024	1.560	14.699	3.021	0.393
β'dock	β': 369-420	52	1.026	0.647	0.479	0.618	1.268	1.392	0.231
β'shelf/β'jaw	β': 787-931, 1135-1317	327	1.710	1.251	1.122	1.073	1.076	2.648	0.462
β'SI3	β': 948-1121	174	2.950	2.339	2.452	1.543	1.554	4.760	0.290
Clamp/ β'dock/shelf/ jaw/SI3/ β'C	β: 1241-1341<br>β': 16-342, 369-420, 787-931, 948-1121, 1135-1373	1037	1.952	1.555	1.346	1.294	8.660	3.071	0.309

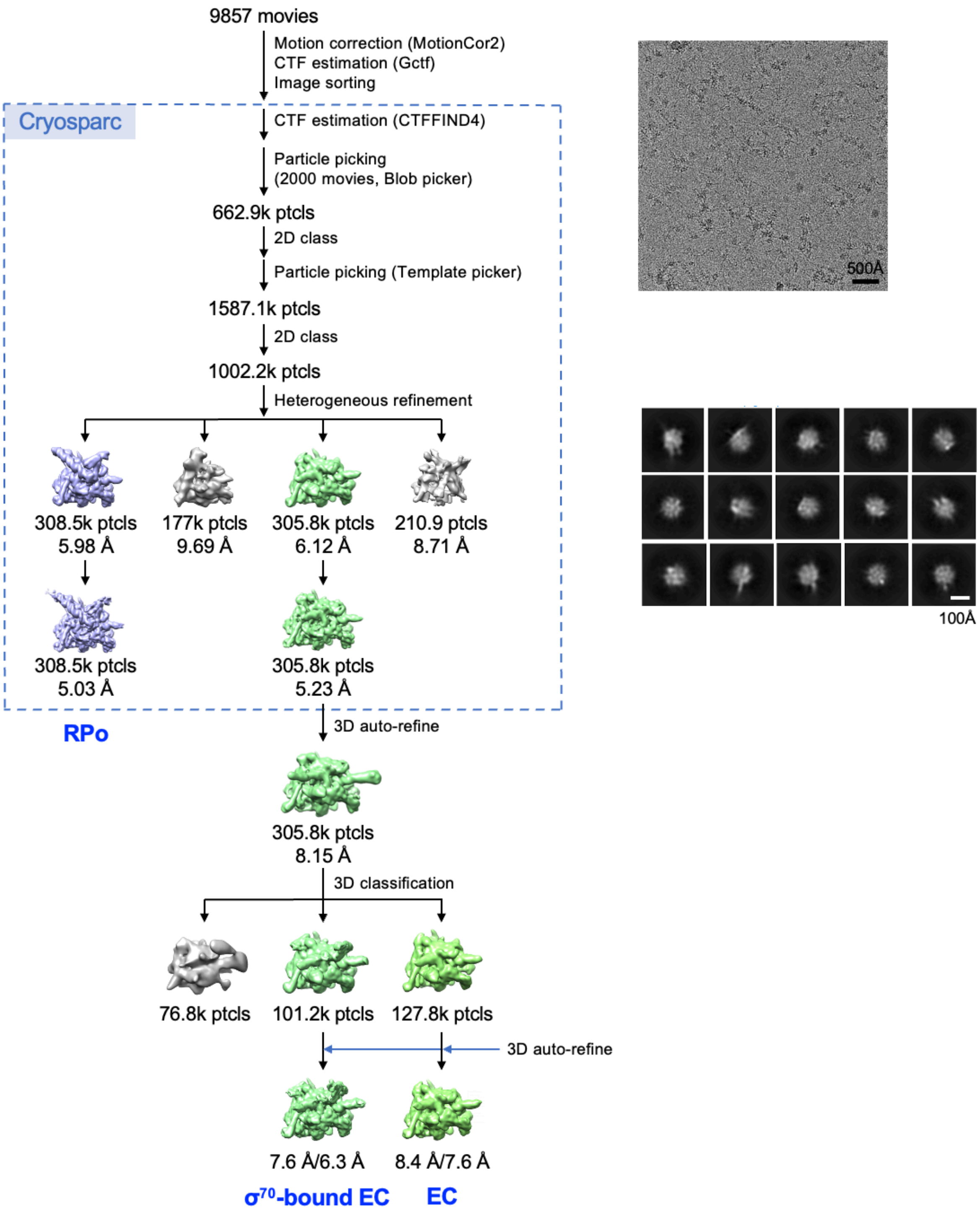

Supplementary Fig. 12 Flowchart for cryo-EM data analysis of *put*-EC. Total 9857 movies of *put*-EC were motion-corrected and summed by MotionCor2[1], CTF-estimated by Gctf[2] and sorted by the CTF parameters in RELION3.1[3]. The sorted images were transferred to cryoSPARC v3.2.0[4] for further process as follows: First, 662.9k particles were picked using blob picker from 2000 movies, extracted with 292 pixels box size, and 2D classified to make picking templates. Then, 1587.1k particles were picked using Template picker from 9820

images. The particles were 2D classified, and 1002.2 k particles from 60 classes were selected. The selected particles were divided into four classes in heterogeneous refinement. As templates for the heterogeneous refinement, RPo, *put*EC, *put*-less EC and σ^{70}-bound *put*EC maps from the *put*EC data were used. Among four classes, two classes were subjected to homogeneous refinement and resulting in an RPo and an EC without intact *put*RNA, which we named '*put*$^-$-EC' to distinguish it from *put*-less EC from the *put*EC data.

All particles of *put*$^-$-EC were imported to RELION3.1 for further refinements. The particles in *put*$^-$-EC class were 3D auto-refined and 3D-classified into three classes. The first class resulted a junk class, and the other two classes were EC with and without σ^{70}, respectively. The population ratio between σ^{70}-bound EC and σ^{70}-unbound EC is about 3:4, which is similar to the ratio between σ^{70}-bound *put*EC and σ^{70}-unbound *put*EC. The particles of σ^{70}-bound EC and EC were 3D auto-refined and postprocessed, yielding 6.3 Å- and 7.6 Å-resolution maps, respectively. For reference, one representative EM image and representative 2D averages are shown beside the pipeline.

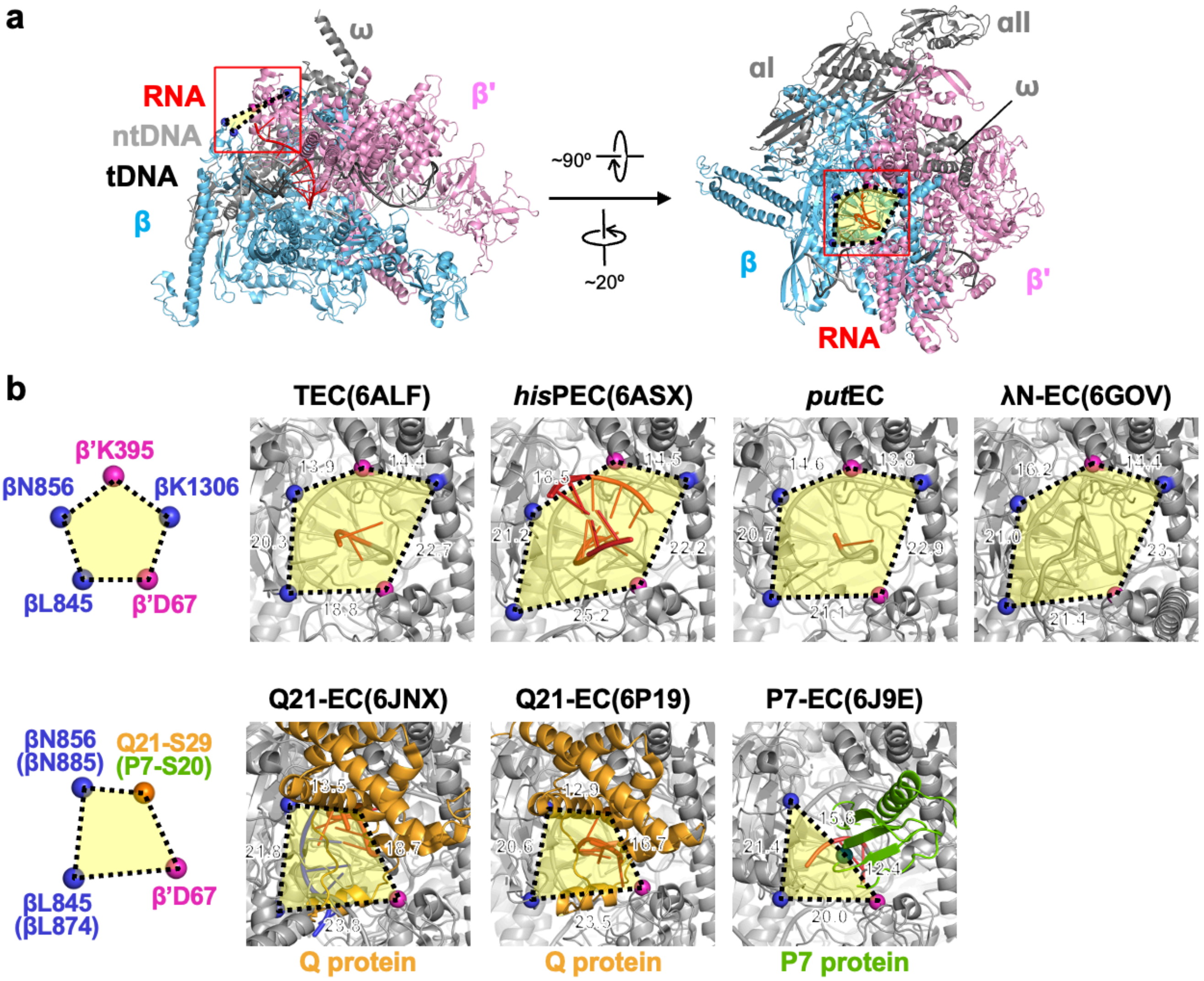

c

	TEC	*his*PEC	*put*EC	λN-EC	Q21-EC (6JNX)	Q21-EC (6P19)	P7-EC
Relative area	1.00	1.16	1.10	1.12	0.66	0.58	0.39

Supplementary Fig. 13 Comparisons of the mouths of the RNA exit channels in anti-termination complexes. a The cryo-EM structure of non-paused EC (PDB 6ALF) is drawn in cartoon format, and the mouth of the RNA exit channel is indicated by dotted lines in a red box. The RNAP subunits are colored as labeled. **b** Close-up views of the RNA exit channel of each anti-termination complex. The mouth of the RNA exit channel is shown by connecting the C_{α} atoms of the five residues of β and β' subunits (shown as colored spheres as in the scheme in the left column) with black dotted lines, and the area is colored in yellow. When the exit channel is restricted by the anti-termination factor, 21Q or P7, a residue from the factor is chosen to re-define the mouth in a rectangular shape. The nascent RNA passing through the exit channel is colored red. The distance between the chosen C_{α} atoms is written in a Å unit. **c** The relative areas of the mouths in **b** are calculated and compared. The *put*EC has a slightly larger size of the channel opening than that of the non-paused EC like *his*PEC and λN-EC, whereas Q21-EC and P7-EC have restricted openings. The RNA exit channel structures of the anti-termination complexes suggest that the Q21 and P7 reduce termination by restricting the RNA exit channel and

interfering with the RNA hairpin formation, which is crucial for termination, while *put*EC and λN-EC utilize different strategies to inhibit transcription termination. The analysis of Qλ-associated anti-termination complex is excluded in this figure because its coordinates is not released yet.

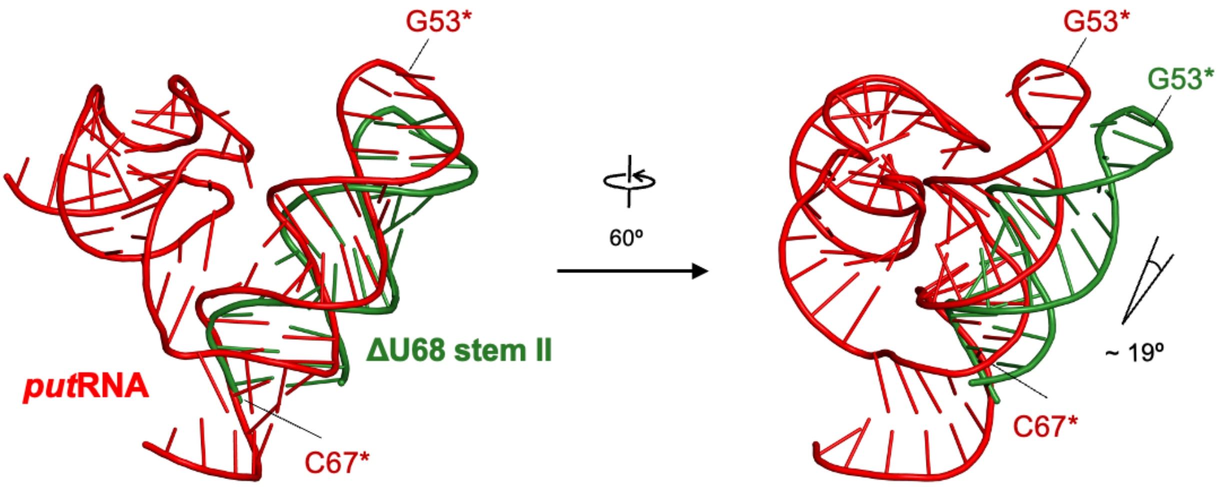

Supplementary Fig. 14 Simulation of HK022 *put*L ΔU68*. To simulate the *put*RNA structure without U68*, a long ideal A-form RNA duplex is superimposed onto the stem II RNA duplex region (G36*-C39*/G69*-C72*) before U68*, and the cleaved *put*RNA stem II region after U68* (G40*-C67*, colored in green) is aligned onto the ideal A-form RNA duplex by its short duplex region (G40*-C41*/G66*-C67*). The template A-form RNA used for the alignment is not shown here for clarity. The angle between the phosphorus atoms of C67* and G53* modeled stem II region was ~19°.

Supplementary Fig. 15 Uncropped scan of a gel from Supplementary Fig. 1b

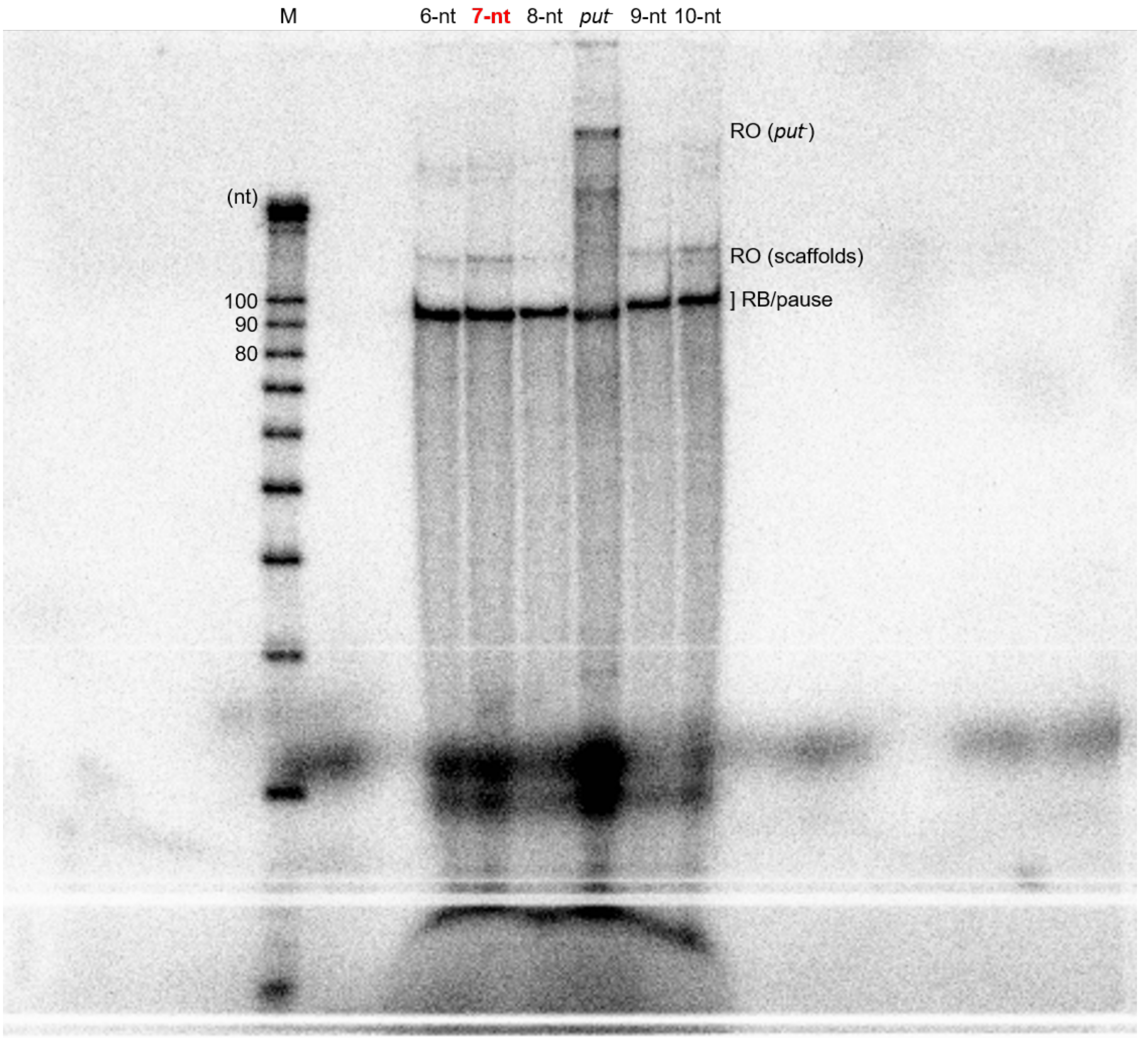

REFERENCES

1. Zheng, S. Q. *et al.* MotionCor2: anisotropic correction of beam-induced motion for improved cryo-electron microscopy. *Nature Methods 2017 14:4* 14, 331–332 (2017).
2. Zhang, K. Gctf: Real-time CTF determination and correction. *Journal of Structural Biology* 193, 1–12 (2016).
3. Scheres, S. H. W. RELION: Implementation of a Bayesian approach to cryo-EM structure determination. *Journal of Structural Biology* 180, 519–530 (2012).
4. Punjani, A., Rubinstein, J. L., Fleet, D. J. & Brubaker, M. A. cryoSPARC: algorithms for rapid unsupervised cryo-EM structure determination. *Nature Methods 2017 14:3* 14, 290–296 (2017).
5. Bepler, T. *et al.* Positive-unlabeled convolutional neural networks for particle picking in cryo-electron micrographs. *Nature Methods 2019 16:11* 16, 1153–1160 (2019).
6. Krissinel, E. & Henrick, K. Inference of Macromolecular Assemblies from Crystalline State. *Journal of Molecular Biology* 372, 774–797 (2007).
7. Will, S., Joshi, T., Hofacker, I. L., Stadler, P. F. & Backofen, R. LocARNA-P: Accurate boundary prediction and improved detection of structural RNAs. *RNA* 18, 900–914 (2012).
8. King, R. A. *et al.* Newly discovered antiterminator RNAs in bacteriophage. *Journal of Bacteriology* 193, 5784–5792 (2011).
9. Kang, J. Y. *et al.* Structural basis of transcription arrest by coliphage HK022 Nun in an *escherichia coli* RNA polymerase elongation complex. *Elife* 6, 1–20 (2017).
10. Kang, J. Y. *et al.* RNA Polymerase Accommodates a Pause RNA Hairpin by Global Conformational Rearrangements that Prolong Pausing. *Molecular Cell* 69, 802-815.e1 (2018).
11. Guo, X. *et al.* Structural Basis for NusA Stabilized Transcriptional Pausing. *Molecular Cell* 69, 816-827.e4 (2018).
12. Abdelkareem, M. *et al.* Structural Basis of Transcription: RNA Polymerase Backtracking and Its Reactivation. *Molecular Cell* 75, 298-309.e4 (2019).
13. Hofacker, I. L. Vienna RNA secondary structure server. *Nucleic Acids Research* 31, 3429 (2003).
