# Supplementary material for: Structural basis of transcriptional regulation by a nascent RNA element, HK022 putRNA
Source: Nat Commun. 2022 Aug 15;13:4668. doi: 10.1038/s41467-022-32315-y (PMC9378689; doi:10.1038/s41467-022-32315-y)
Supplement: Supplementary file 3 — Reporting Summary [file 41467_2022_32315_MOESM3_ESM.pdf]

nature portfolio

Corresponding author(s): Jin Young Kang

Last updated by author(s): Jul 13, 2022

Reporting Summary

Nature Portfolio wishes to improve the reproducibility of the work that we publish. This form provides structure for consistency and transparency in reporting. For further information on Nature Portfolio policies, see our Editorial Policies and the Editorial Policy Checklist.

Statistics

For all statistical analyses, confirm that the following items are present in the figure legend, table legend, main text, or Methods section.

n/a	Confirmed	
☐	☒	The exact sample size (*n*) for each experimental group/condition, given as a discrete number and unit of measurement
☐	☒	A statement on whether measurements were taken from distinct samples or whether the same sample was measured repeatedly
☒	☐	The statistical test(s) used AND whether they are one- or two-sided<br>*Only common tests should be described solely by name; describe more complex techniques in the Methods section.*
☒	☐	A description of all covariates tested
☒	☐	A description of any assumptions or corrections, such as tests of normality and adjustment for multiple comparisons
☐	☒	A full description of the statistical parameters including central tendency (e.g. means) or other basic estimates (e.g. regression coefficient) AND variation (e.g. standard deviation) or associated estimates of uncertainty (e.g. confidence intervals)
☒	☐	For null hypothesis testing, the test statistic (e.g. *F*, *t*, *r*) with confidence intervals, effect sizes, degrees of freedom and *P* value noted<br>*Give P values as exact values whenever suitable.*
☒	☐	For Bayesian analysis, information on the choice of priors and Markov chain Monte Carlo settings
☒	☐	For hierarchical and complex designs, identification of the appropriate level for tests and full reporting of outcomes
☒	☐	Estimates of effect sizes (e.g. Cohen's *d*, Pearson's *r*), indicating how they were calculated

Our web collection on statistics for biologists contains articles on many of the points above.

Software and code

Policy information about availability of computer code

Data collection	EPU2.6
Data analysis	Thermo Xcalibur Qual Browser (version 4.2.47) and UniDec (version 4.2.0) were used for native MS analyis. MotionCor2 (v1.4), RELION 3.1, Gctf, CryoSPARC (v.3.2.0), bsoft package (v2.0.5), UCSF Chimera (v1.11.12), PHENIX (version 1.18.2), and PyMOL (version 2.4.0) were used for cryo-EM data analysis, model building, and structure visualization

For manuscripts utilizing custom algorithms or software that are central to the research but not yet described in published literature, software must be made available to editors and reviewers. We strongly encourage code deposition in a community repository (e.g. GitHub). See the Nature Portfolio guidelines for submitting code & software for further information.

Data

Policy information about availability of data

All manuscripts must include a data availability statement. This statement should provide the following information, where applicable:

- Accession codes, unique identifiers, or web links for publicly available datasets
- A description of any restrictions on data availability
- For clinical datasets or third party data, please ensure that the statement adheres to our policy

Cryo-EM data have been deposited in the RCSB Protein Data Bank (www.pdb.org) and in the Electron Microscopy Data Bank (www.emdatabank.org). The accession number for the coordinate and cryo-EM map for putEC are 7XUE and EMD-33466, respectively. The accession number for the coordinate and cryo-EM map for put-less EC are 7XUG and EMD-33468, respectively. The accession number for the coordinate and cryo-EM map for σ70-bound putEC are 7XUI and EMD-33470,

respectively.

Human research participants

Policy information about studies involving human research participants and Sex and Gender in Research.

Reporting on sex and gender	Not applicable
Population characteristics	Not applicable
Recruitment	Not applicable
Ethics oversight	Not applicable

Note that full information on the approval of the study protocol must also be provided in the manuscript.

Field-specific reporting

Please select the one below that is the best fit for your research. If you are not sure, read the appropriate sections before making your selection.

[x] Life sciences [ ] Behavioural & social sciences [ ] Ecological, evolutionary & environmental sciences

For a reference copy of the document with all sections, see nature.com/documents/nr-reporting-summary-flat.pdf

Life sciences study design

All studies must disclose on these points even when the disclosure is negative.

Sample size	For cryo-EM study, we collected 8174 movies in a single cryo-EM session. This dataset provided more than 1 million particles, and they are sub-classified into four classes. Each class contains similar to or more than 100k particles and resulted in a decent map having sub-4A resolution. This satisfies a conventional criteria for cryo-EM map reconstruction.
Data exclusions	During cryo-EM data analysis, we performed heterogenous refinement in cryoSPARC or 3D classification in RELION. Among the resulting classes, the classes that have poor map density were excluded because the particles in the classes are possibly damaged protein complexes, contaminants, or mis-picked background.
Replication	Each class contains > 88,700 particles. Because each class is reconstructed with this vast number of particles reaching to high-resolution, each structure itself is replicated enough.
Randomization	Randomization is not applicable to our study because the kind of the sample is in vitro protein assembly captured in a thin ice. Because we collected cryo-EM images on a cryo-EM grid area with optimal ice thickness and cannot choose specific set of particles during data collection, randomization is already done during the data collection process. In addition, the number of particles used in the data analysis exceeds one million, therefore, the dataset itself is already randomized enough.
Blinding	Blinding is not applicable in our study. In the data analysis, data processing software classifies the data to see the conformational differences in the protein complex population. Therefore, we cannot interfere the data process, and therefore, already are blinded on the data analysis.

Reporting for specific materials, systems and methods

We require information from authors about some types of materials, experimental systems and methods used in many studies. Here, indicate whether each material, system or method listed is relevant to your study. If you are not sure if a list item applies to your research, read the appropriate section before selecting a response.

Materials & experimental systems

n/a	Involved in the study	
[x]	[ ]	Antibodies
[x]	[ ]	Eukaryotic cell lines
[x]	[ ]	Palaeontology and archaeology
[x]	[ ]	Animals and other organisms
[x]	[ ]	Clinical data
[x]	[ ]	Dual use research of concern

Methods

n/a	Involved in the study	
[x]	[ ]	ChIP-seq
[x]	[ ]	Flow cytometry
[x]	[ ]	MRI-based neuroimaging
